# Emotion Analysis of Cross-Media Writing Text in the Context of Big Data

**DOI:** 10.3389/fpsyg.2022.835149

**Published:** 2022-04-13

**Authors:** Rui Ren

**Affiliations:** College of Teacher Education, East China Normal University, Shanghai, China

**Keywords:** emotion analysis, big data, cross-media writing, text feature, natural language processing

## Abstract

Since the beginning of the 21st century, sentiment analysis has been one of the most active research fields in natural language processing. Now sentiment analysis technology has not only achieved significant results in academia, but also has been widely used in practice. From business services to political campaigns, sentiment analysis is used in more and more fields. Sentiment analysis is essentially to dig out the user’s emotional attitude from the massive emotional natural language text data, and analyze the emotional dynamics of the text author through certain technical means. At present, there is almost no sentiment analysis in cross-media writing content, and it can rarely help cross-media writing vision to advance with the times and comprehensive improvement of writing ability to adapt to the current rapidly developing information society; the commonly used text media in the digital age are not. Then there is the only composition tool. Various new media appearing with the development of the ages continue to intervene in writing, and it is the general trend to cultivate media literacy in writing. The main content of this paper is the research on the emotional intensity of students’ Internet public Aiming at the shortcomings of the topic feature word selection in the sentiment tendency analysis of the students’ Internet public opinion, improvements have been made to facilitate the research on the sentiment intensity of the sentiment analysis of the students’ Internet public opinion. Sentiment analysis of students’ cross-media written text through an improved MapReduce combinator model.

## Introduction

Along with the advance of social economy and technology, all sorts of mature technology has begun to infiltrate into all aspects of the social realm, the expansion of network information technology, video media, news media, community, BBS community, all kinds of network environment has become the main place of multimedia resources promotion, special education media, news media, mobile digital media arises at the historic moment. Faced with how to choose a large number of data resources, the audience will inevitably be at a loss, and the comment section has become the main channel for the audience to understand the status of resources. Semantic analysis refers to the establishment of analysis models for various information text resources. These information text resources include online teaching courses, news media, students’ forum discussions and digital compositions. By 2021, China’s Internet penetration rate had reached 71.6 percent. There is a wealth of information on cross-media writing resources on the Internet (Deshpande, 2004). If the method of combining media literacy education with writing teaching is really applied to writing teaching practice in China, it can not only cultivate students’ writing ability, media learning and use skills, but also improve their thinking ability, problem-solving ability, constructive learning and lifelong learning ability. How to effectively and quickly analyze and evaluate the above-mentioned text resources and then integrate the above-mentioned information text resources has become one of the urgent problems to be solved in the era of information explosion (Bala et al., 2014; Dewangan et al., 2016; Fang et al., 2016; Wang et al., 2016; Sébastien and Lecron, 2017). Based on big data technology, this paper establishes a cross-media written text sentiment analysis model to analyze cross-media written texts. Through the result test, the established model has good applicability.

## Related Theoretical Methods

### Big Data Technology

How to rapidly process media written data and extract effective information, the requirements of processing technology are also increasing. The data processing technology of big data technology in media writing is also developing rapidly, whether it is the processing, identification and analysis of cross-media data, it can be well applied. As for the definition of big data, different organizations have given different definitions, but they all focus on the characteristics of large data scale, fast data circulation, huge data categories, and difficulty in extracting effective information. The significance of big data lies not in the collection and storage of data, but in how to handle such a large data set, how to extract valid data from fast-moving data, and how to organize, analyze and predict the data. Currently, Hadoop and Spark are widely used big data processing tools. Hadoop stores a large amount of data on multiple node servers to realize distributed storage of data. The built-in MapReduce in Hadoop can perform large-scale distributed processing of distributed stored data, but the processing speed cannot be compared with Spark. Spark also provides a SparkSQL database for interactive data query, and a variety of machine learning algorithms. MLlb library (Dahiwale et al., 2014).

### Cross-Media Text Natural Language Processing

#### Syntactic Analysis

As a shallow semantic analysis, syntactic analysis is widely used in natural language processing. The main method is to rely on parsing. The main task of dependency parsing is to analyze the structure of sentence components and determine the dependency relationships between phrases or words. The syntactic structure of sentences is obtained by analyzing the dependency relation of sentences. Subordinate syntax maintains that the core word of the sentence is the verb, and other words dominate. Thus, in a relationship, the other components belong to the verb, and the verb is the dominant, so it is not dominated by the dominant. The conditions for dependency parsing are as follows (Kim and Lee, 2002; Martínez et al., 2017; Zenebe and Norcio, 2019):

(1) The independent component in the sentence is unique.
(2) The other elements in the sentence, that is, the dominated person, are all subject to a certain dominance.
(3) Any subject in the sentence can only depend on the only one dominator.
(4) If the component P is directly subordinate to another component Q, and the position of the component S in the sentence is between the components P and Q, then the component S is subordinate to the component P or to the component Q, or to the component A component between P and Q.
(5) The components on the left and right sides of the central component of the sentence do not have any relationship with each other.

**FIGURE 1:**
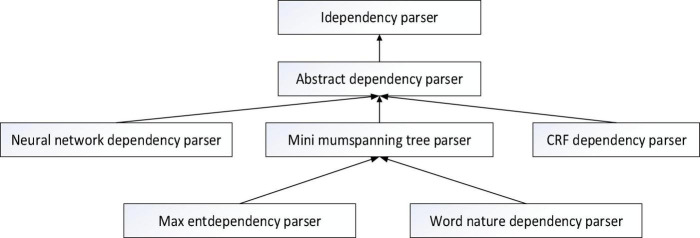
Class diagram of the syntactic analyzer.

The syntactic parsing tool is staford-Parser and Hanlp toolkit developed by Stanford University Natural Language Research and Development team. It is based on Java, easy to operate, scalable and supports many languages. Parsing is done by calling interfaces in packages. Internally use neural networks to rely on parsers. Users can invoke the Neural Network Dependency directly Parser.compute (sentence) or column search by calling ArcEager transport system relies on KBeamArcEagerDependency-Parser to implement parsing function. The syntactic analysis tool used in this study is based on big data technology, and the Hanlp tool of syntactic analysis is used to analyze the syntax. The analyzer class diagram is shown in Figure 1.

#### Part-of-Speech Tagging

Part of speech tagging is one of the core contents of data extraction in cross-media writing. By labeling the words in the sentences of *trans*-media written texts, selecting the relationship between different parts of speech and formulating corresponding extraction rules, the target text data can be extracted. Corpus-based natural language processing has been widely used in many kinds of natural language processing. The learning method of pos tagging enables natural language to be expressed in a form that is very easy to understand. Big data and sentiment analysis can be used to quickly analyze the emotions expressed in media writing texts. The accuracy of pos tagging directly affects the effect of sentiment mining of text data. Improving the quality of pos tagging is an effective way to improve the efficiency of text emotion mining. The transform-based error driven machine learning method can successfully detect the pos tagging error location and generate errors. Fixing the rules helps to correct errors. In view of different error situations, rules that distinguish context, context-sensitive or context-independent, are proposed to realize the function of automatic error correction (Sarwar et al., 2002; Linden et al., 2003; Frémal and Lecron, 2017).

### Parallel Programming Model MapReduce

#### MapReduce Programming Model

**FIGURE 2:**
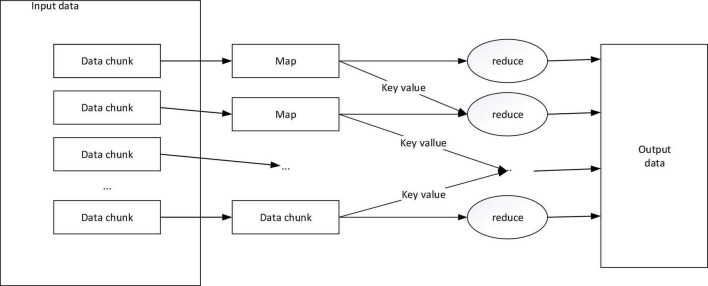
MapReduce calculation process.

The MapReduce model divides the calculation process into a Map phase and a Reduce phase. In the Map task, the input data is a piece of cross-media writing text, each document can be regarded as an element, and each data block can be regarded as a collection of multiple elements, and the same document It is not possible to store across data blocks. And in the model, all input and output data forms are based on key-value pairs, which is to facilitate the combined use of the model. The specific calculation process of the MapReduce program is shown in Figure 2.

The Map task is to convert the input information into an intermediate key-value pair, where the key value is not unique and repeatable, and then use the MapReduce framework to classify and summarize all intermediate key-value pairs generated by the Map process by key value OK, upload it to the Reduce process as input. The Reduce task is to accept the key value and the corresponding set of value values, and recalculate and merge to get the value or key value we need (Hinton, 1986; Taboada et al., 2011; Turney, 2020).

#### MapReduce Execution Process

**FIGURE 3:**
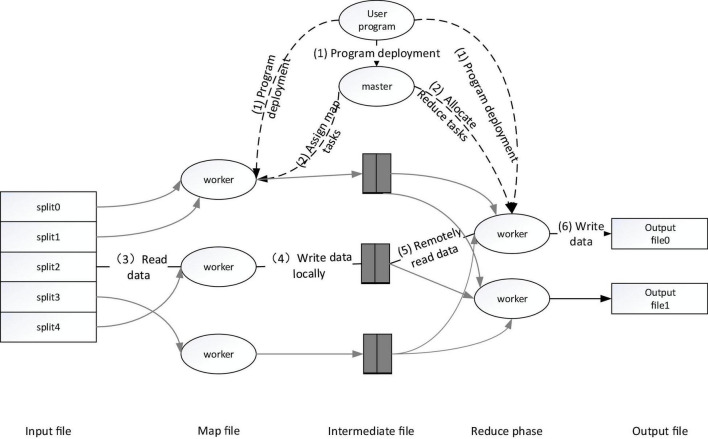
Execution process of MapReduce operation.

The execution flow of the MapReduce operation is shown in the figure below. When the user requests to call the MapReduce function, the executed process is shown in Figure 3.

(1) First, slice the input cross-media writing text, which can be divided into M data blocks, each data block is usually 16–64 MB, and then use fork to copy the user process to other machines in the cluster (Kim and Lee, 2002; Turney and Littman, 2002; Mikolov et al., 2013; Turney, 2020).
(2) The master is responsible for scheduling, assigning work to idle workers, and executing Map tasks or Reduce tasks.
(3) After the worker is assigned the Map task, it starts to read the input media and write text data block fragments. The Map task extracts key-value pairs from the input data, passes the key-value pairs as input parameters to the map function, and gets the middle The key-value pairs are cached in memory.
(4) The worker executes the Map task, and the obtained cached intermediate key-value pairs will be periodically saved locally, partitioned, and then corresponding to the Reduce task.
(5) The master schedules the worker to execute the Reduce task, and the reduce worker reads the output file of the map task, reads the corresponding intermediate key-value pair information, sorts it, and gathers the same key-value pairs together.
(6) The reduce worker passes each key and corresponding value to the Reduce function as input according to the key-value pairs obtained in the previous step, and then saves the output of the Reduce task obtained in HDFS.

MapReduce technology is a parallel computing model, which solves some important problems such as fault tolerance and scalability at the system level. Through user-written Map and Reduce functions, the massive media writing text data can be operated in parallel on a large-scale cluster. It can be processed and analyzed quickly and efficiently.

### Sentiment Analysis of Cross-Media Writing Text

Sentiment analysis is the core content of sentiment orientation mining in Chinese corpus and an important basis for judging sentiment orientation. Emotion analysis is one of the main applications in *trans*-media writing. Words with emotional polarity. Emotional polarity words refer to words or texts with emotional tendencies. At the same time, these words are often modified by some modifiers. These modifiers are called adverbs of degree, such as “very” and “very” and so on. Chinese corpus sentiment analysis is to study these sentiment words and their modifiers in the text. Emotional words are usually positive words with positive meaning and negative words with negative meaning. Adverbs of degree can enhance or weaken the polarity of emotional words. The research content of this topic is mainly *trans*-media writing data, mainly sentence-based sentiment analysis. At present, the research methods using sentence as emotion analysis unit are mainly based on machine learning, semantic grammar and emotion dictionary. This article mainly uses a dictionary-based approach.

## The Construction of a Semantic-Based Cross-Media Writing Text Emotional Intensity Model

### Text Preprocessing for Cross-Media Writing

For the text preprocessing in the media writing text, it is mainly word segmentation, part-of-speech tagging, and filtering of useless words, in order to be able to transform the most primitive text content into a mathematical model to represent. Chinese word segmentation is different from English word segmentation, and has rich semantics, which is easy to cause ambiguity. Therefore, in an article, the accuracy of the word segmentation is a prerequisite, which will directly affect the result of a series of subsequent processing.

Part-of-speech tagging is the process of determining and marking the appropriate part-of-speech according to the meaning of words in an article and the context of the context. Because the same word may have multiple parts of speech, it is necessary to uniquely determine the part of speech of a vocabulary and label it, not only by relying on the vocabulary itself, but also by taking into account the context to eliminate the ambiguity of the vocabulary labeling, so as to select the most appropriate one Part of speech is used as the tagging result. Useless words refer to meaningless words that appear frequently in the text but basically have no effect on the distinction of the text. Words such as “of” and “yes” are all useless words and need to be filtered from the text collection.

### MapReduce-Based Cross-Media Writing Text Feature Word Extraction Algorithm

**FIGURE 4:**
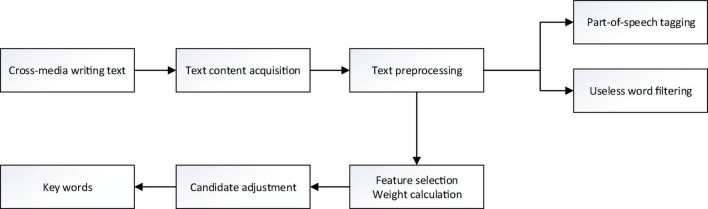
The main steps of keyword extraction.

This article uses the ICTCLAS word segmentation system to segment the obtained media writing text and mark the part of speech. First, it will include the core topic information, and the vocabulary form of the media writing text is relatively large, but only a few words are needed to express the medium. The theme to be reflected in the text is the key word that reflects the theme of the text. Figure 4 shows the main steps of extracting keywords.

#### Feature Selection and Weight Calculation

Feature selection refers to selecting a small part of the most effective features from the original feature set according to certain rules to represent the whole feature set, which can be understood as selecting a relatively optimal subspace from the original high-dimensional feature space. The focus of feature selection research on text data is the evaluation function used to measure the importance of words. The process is to first calculate the importance value of each word according to the evaluation function, and then select all the words whose value exceeds the threshold according to the preset threshold. The existing feature selection methods, such as document frequency method, information gain method, mutual information method and other dimensionality reduction methods, only select part of the representative feature items from the feature item set, that is, the subset of the original feature item dictionary. In this paper, term frequency-inverse Document frequency (TF-IDF) method based on the improved MapReduce model is used to calculate the term statistics and weights of preprocessed media written texts.

#### Improved MapReduce Combiner Model

**FIGURE 5:**
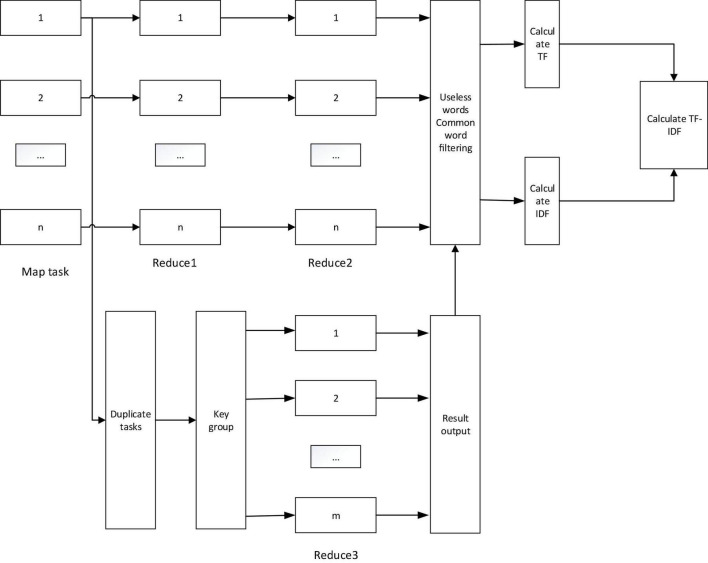
Work flow chart of the improved MapReduce model.

It is obtained through the Reduce function. Here we need to improve the MapReduce model. Therefore, we have made improvements on the basis of the original MapReduce, and nested the Map and Reduce tasks in MapReduce to form a MapReduce combinator. The workflow of the improved MapReduce model is shown in Figure 5.

Among them, the Map task is to convert the input information into a sequence of intermediate key-value pairs; the Reduce1 task is to count the number of a word in a document; the Reduce2 task combines all the keys in all single media documents and calculates all The sum of all calculated values in the Reduce1 task is the sum of the number of occurrences of all words in a single media document; the deduplication task is to set the value to 1, which is used to calculate the number of words appearing in a document to prevent Recording multiple times has an impact on the result; key grouping is to merge all the key-value pairs of the same key into (k,[v_1_, v_2_,…, v_n_]), and then use it as the input of the Reduce3 task; Reduce3 The task is to add the value corresponding to the key value to calculate the number of words contained in the document; the final filtering step of useless and common words is to filter some words that do not affect the result or have a negligible effect.

### Construction of a Sentiment Analysis Model for Cross-Media Writing Text

Through the above improved MapReduce model, the processed network data is finally obtained. For all cross-media writing content, a high-dimensional sparse matrix is obtained. According to a given threshold, the first 10 TF-IDF values in the medium writing text are retained., The topic can be identified accordingly, but if you want to measure the public opinion index reflected by the topic, it is impossible to rely on the TF-IDF index alone. The number of media written texts corresponding to the topic can be used as the measurement of the public opinion index.

Since the number of corresponding media writing texts can be obtained according to the similarity of the title, in this article, the method of matrix and vector multiplication based on the MapReduce model is used for calculation. First, the topic and the document are expressed in the form of a vector space model (VSM) Information corresponds to the rows and columns of the VSM, and then take the product of the VSM and the unit column vector to get the number of media writing texts corresponding to the topic, but the dimension of the vector should be determined according to the actual data information. Since the correlation vector of the VSM is generated after a series of operations from the massive network data, its dimension is completely beyond the scope of traditional calculation methods, so we adopt the matrix-vector multiplication based on the MapReduce model.

The implementation process of matrix-vector multiplication based on the MapReduce model is: suppose that the matrix M is m × n dimensional, and the element m_ij_ in the matrix M is used to represent the element in the i-th row and j-th column, and there are n-dimensional column vectors V, vector The element v_j_ in V represents the jth element. Therefore, the product of the matrix M and the column vector V can be represented by an m-dimensional column vector X, and the i-th element in the vector X is:


(1) $$x_{i}=\sum_{j=1}^{n}m_{ij}v_{j}$$

Among them, as in formula (1), for the element m_ij_ in the matrix M, the key-value pair after the Map task output is (i, m_ij_), and then multiplied by the column vector V to obtain n m_ij_v_j_. It can be seen that the key value is the same, and the result obtained by combining the key value by the MapReduce framework is used as the input of the Reduce task, and then after the addition operation, the n m_ij_v_j_ are added to obtain (i, x_i_). Therefore, the final output of Reduce is the vector X. The larger the proportion of X, the stronger the sentiment of the text.

## Test and Results Analysis

### Algorithm Description

#### Sentiment Analysis Process

(1) Text segmentation conversion. Regarding the cross-media writing text of this article, the written text is regarded as the largest analysis object, and the smallest analysis object is a single sentence. A single sentence is divided into texts by using a Chinese word segmentation tool as a separator. For a single sentence, the text is divided into single sentences in order to obtain a format that can be easily analyzed later, for example, the sentence “I am very unhappy today.” After word segmentation, it can be converted to:
  [(1, “I,” “r”), (2, “today,” “t”), (3, “very,” “d”), (4, “no,” “d”), (5, “Happy,” “a”)].
(2) Emotional positioning. According to the above-mentioned sentiment dictionary constructed based on the existing relatively mature Chinese sentiment dictionary, the divided words of each sentence are queried one by one with the sentiment vocabulary in the constructed sentiment dictionary. If it can be found, it can be confirmed as a sentiment word and obtained To the corresponding emotional polarity and emotional intensity value, record the result as (position in the sentence, emotional polarity, emotional intensity value), if it does not exist, it is not an emotional word, and so on, until all words in a single sentence are The traversal is complete and ends. For sentiment analysis in the text, it is analyzed based on the sentiment words in the sentence, and the sentiment of the sentence is calculated by the sentiment polarity and intensity of the sentiment word, thereby determining the sentiment of the entire text.
(3) Emotional integration. Emotional inclination can be calculated by integrating the emotional inclination of all single sentences according to certain rules. The emotional tendency of a sentence is calculated from the emotional words contained in the single sentence and their modifications. After the first two steps of operation, the division of single sentences can be obtained, as well as the emotional words, negative words and degree adverbs in each sentence and the corresponding emotional intensity value. When calculating the emotional tendency of a single sentence, the syntactic structure needs to be considered. The so-called syntactic structure analysis of a single sentence is to analyze the relationship between related words in the sentence and the structure of the sentence, and carry out related processing. First, use the results obtained by the syntactic analysis system as a reference to extract the structure of the sentence, mainly including emotional words, modifiers, etc., to determine the relationship between each word. Through syntactic analysis, the structure of the sentence is judged and the relationship between key words is obtained for sentiment calculation and analysis.

(1) When there are no modifiers in the sentence. In this case, it shows that the polarity and strength of emotional words determine the emotional tendency of emotional sentences.
(2) When the sentence contains modifiers. This article mainly considers the modification of degree adverbs and negative words. Adverbs of degree can strengthen and weaken the strength of subjective words in sentences, while negative words can completely reverse the polarity of emotion words and opinion words.
  (a) The treatment of adverbs of degree. Match the word segmentation results containing the modified sentence with the degree adverbs in the sentiment dictionary. If the matching is not successful, the sentiment polarity of the target sentiment word remains unchanged; if the matching is successful, it will be marked according to “Modern Chinese: A Study of Degree Adverbs” The intensity of the target emotional word adjusts the emotional polarity of the target emotional word. In this study, adverbs of degree are divided into 4 levels according to different degrees. Words such as “very, extremely” are defined as the highest level, with a weight coefficient of 2, and words such as “comparative and slightly” are defined as In the second level, the weight coefficient is 1.5. Words such as “return and barely” are defined as the third level, and the weight coefficient is 0.5. For target emotional words without degree word modification, the default degree weight coefficient is 1, that is The emotional strength value of the target emotional word itself, therefore, the polarity of the emotional sentence remains unchanged, and the emotional strength is obtained by the product of the modifier weight coefficient and the emotional strength of the emotional word, that is, the emotional value of the emotional sentence = the weight of the degree adverb Coefficient × emotional polarity value of emotional word × emotional strength of emotional word.
  (b) Treatment of negative words. The word segmentation result containing the modified sentence is matched with the negative word in the sentiment dictionary. If the matching is not successful, the sentiment polarity of the target sentiment word remains unchanged; if the matching is successful, the following conditions are considered. If the negative word is the negation of another negative word, then it means double negation, that is, it means affirmation, then the emotional polarity remains unchanged; if the negative word is the negative of the target emotional word, then the emotional polarity of the emotional word Reverse. Therefore, the emotional strength of the emotional sentence remains unchanged, while the emotional polarity will change accordingly, that is: the emotional value of the emotional sentence = the weight coefficient of the negative word (−1) × the emotional polarity value of the emotional word × the emotional strength of the emotional word.

#### Emotion Intensity Calculation Model

Sentiment analysis can be regarded as a subjective evaluation or an inherent preference tendency of the judgment subject to the judgment object. There are two main dimensions of emotional orientation: one is emotional polarity, and the other is emotional strength. In sentiment analysis, in order to highlight differences, each sentiment word is usually given a different weight, so as to better perform sentiment analysis on the text.

(1) The intensity of objective emotions. Objective emotions mainly express emotional tendencies through emotional words in the text. For the emotional words in the target emotional sentence, the emotional value of the sentence *O*_*S*_*i*_ can be quantitatively expressed through the above rules. Therefore, the calculation formula for the internal emotion strength of the text T_i_ containing n sentences is shown in formula (2).

(2) $$O\_T_{i}=\frac{\sum_{i=1}^{n}\eta \times O\_S_{i}}{n}$$

In the formula, η represents the influence factor of emotional words in the text. O_si_ represents the emotional strength of the emotional sentence S_i_.

(2) Subjective emotional intensity. The intensity of subjective emotion mainly refers to the degree of attention to the text of cross-media writing. As far as news text is concerned, the degree of attention is mainly determined by its sharing and reply value. Assuming that the related replies of the same cross-media text are independent of each other, the calculation formula of subjective emotion strength is shown in formula (3):

(3) $$S\_T_{i}=\frac{\sum_{i=1}^{n}\log\left(1+\frac{2T_{share}T_{reply}}{T_{share}+T_{reply}}\right)}{n}$$

In formula (6), T_share_ represents the amount of forwarding and sharing of cross-media writing texts, and represents the amount of replies to cross-media writing texts.

(3) The overall emotional intensity. The overall emotional intensity of the event is determined by the combination of objective emotional intensity and subjective emotional intensity, which can be calculated by formula (4):

(4) $$x_{i}=\frac{a*O\_T{+}_{i}+\beta *S\_T_{i}}{\alpha +\beta }$$

Therefore, according to the final result, the emotional polarity and emotional strength of the content embodied in the target text can be obtained.

### Evaluation Criteria for Test Results

The experiments in this chapter mainly use evaluation indicators that are accuracy (Precision), recall (Recall), and *F*-measure (*F*-Measure) to measure the accuracy and effectiveness of these two indicators. The corresponding indicators in the evaluation of feature extraction and weight calculation can be defined as:

(1) Precision:

Precision = The extracted effective feature words/the total number of all extracted words in the text.

(2) Recall rate:

Recall = The extracted effective feature words/the total number of words in the text.

The corresponding indicators in text sentiment analysis can be defined as:

(1) Accuracy (precision):

Precision = (The number of correct texts in the test data set to determine the positive and negative tendencies of the text/The number of texts in the test data set to determine the positive and negative tendencies of the text) × 100%.

(2) Recall rate (recall):

Recall = (The number of correct texts in the test data set to determine the positive and negative tendencies of the text/the number of texts in the test data set that actually have positive and negative tendencies) × 100%.

Then the expression of *F*-value can be expressed as:


$$F-value=\frac{2\times precision\times recall}{precision+recall}\times 100\%$$

The evaluation index results mentioned are all reserved with 2 significant digits.

### Test and Result Analysis

#### Feature Selection and Weight Calculation of Text Subject Terms

This paper selects 30 cross-media writing texts. The method based on the TF-IDF index and the improved TF-IDF index method based on the MapReduce combiner are used to compare the effect of text topic word recognition. The experimental results of the two methods are shown in Tables 1, 2.

**TABLE 1:** Experimental results based on TF-IDF method.

Number of text subject words	Accuracy rate	Recall rate	*F*-value
10	0.47	0.42	0.46
9	0.43	0.52	0.48
7	0.40	0.44	0.40
4	0.30	0.39	0.36

**TABLE 2:** Experimental results of the improved method.

Number of text subject words	Accuracy rate	Recall rate	*F*-value
10	0.77	0.68	0.72
9	0.74	0.76	0.75
7	0.70	0.58	0.63
4	0.63	0.70	0.71

Through the comparison of the data in the above two tables, it can be seen that the improved method in the text is more effective than the ordinary TF-IDF method for calculating the feature weight of the topic word, which is beneficial to the calculation and extraction of the topic word, and the same in the extraction in the case of subject headings, evaluation indicators such as accuracy rate and recall rate have relatively good effects.

**FIGURE 6:**
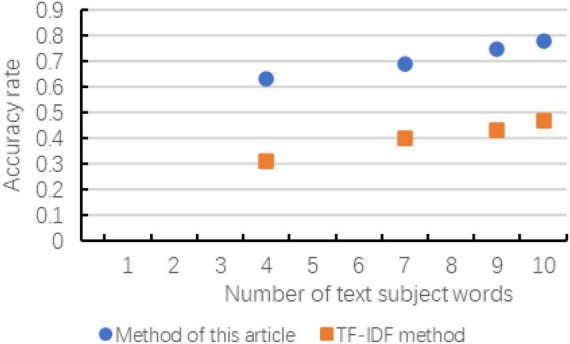
Changes in accuracy of the comparison of the two methods.

**FIGURE 7:**
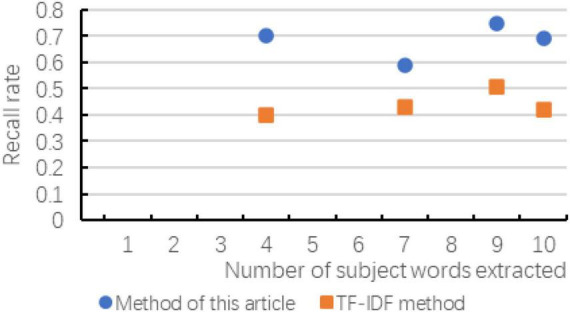
The change of recall rate compared with the two methods.

**FIGURE 8:**
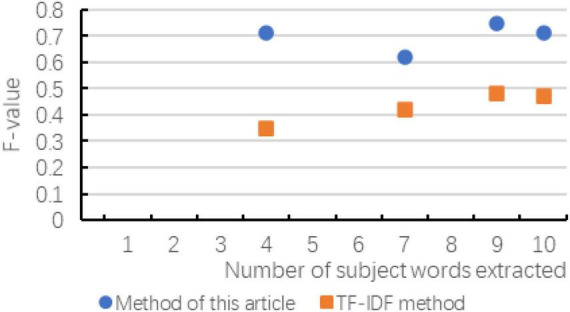
*F*-value changes compared with the two methods.

According to the experiment in Chapter 3, the following result diagrams obtained mainly reflect the changes of the evaluation indicators of the two methods with the number of extracted subject words, and the changes in accuracy, recall and *F*-value are shown in Figures 6–8 show:

According to the above chart, it can be known that when the number of subject terms extracted in cross-media writing is the same, the method in this paper exceeds the TF-IDF method in terms of accuracy, recall and *F*-value, and the trend of the curve is basically with the subject term. The increase in the number has improved the effect. Although in the recall rate and *F*-value chart, when the number of subject words extracted is small, there is a possibility of decline, but as the number of subsequent increases, the effect is more significant. As can be seen in the figure, as the number of topic words extracted first increases and then slows down, indicating that the number of topic words extracted is not as large as possible. Although the accuracy rate is improving, the recall rate and *F*-value have not been consistent. It is improving. Therefore, considering the overall consideration, the number of subject terms is not as good as possible. It is better to choose between 8 and 12.

#### Analysis of Text Sentiment Tendency

In this experiment, the collected cross-media writing text data set was used to conduct the experiment. Three types of texts, including health, education, and culture, were selected in the data set, and 30 positive and negative texts were included as the test data for this experiment. In the first set of experiments, the Chinese sentiment vocabulary ontology database was used, in the second set of experiments, the HowNet dictionary was used, and in the third set of experiments, the sentiment dictionary constructed by the combination of the two was used for testing. The results are shown in Table 3.

**TABLE 3:** Results of analysis of text orientation.

Text subject category	Accuracy rate	Recall rate	*F*-value
Category one (Health)	0.64	0.58	0.59
Category two (Education)	0.70	0.65	0.68
Category three (Cultural)	0.80	0.74	0.77

It can be seen from the experimental results that comparing the test results of the three sets of experiments, using the new sentiment constructed by the Chinese sentiment vocabulary ontology database and the HowNet dictionary to analyze the text orientation is better than using the sentiment dictionary alone to judge the positive and negative orientation of the text. The accuracy rate, recall rate and *F*-value have been improved. Therefore, it is advisable to use the sentiment dictionary constructed in this article when analyzing the sentiment intensity of news text.

## Conclusion

Natural language processing is the basic work of artificial intelligence, including speech recognition, machine translation, public opinion analysis and many other branches. With the rapid development of Internet information technology, especially the continuous maturity of big data analysis technology, we should see that sentiment analysis in the field of Tibetan language has a weak foundation and a late start. The corpus is not perfect, and all aspects of work need to be improved urgently. There is a large space for research.

Firstly, this paper studies and improves the extraction method of text subject words. In view of the deficiency of traditional technology in processing *trans*-media written text data and the characteristics similar to big data, the core technology MapReduce model is improved to mine *trans*-media written text, and Map and Reduce functions are combined into a combinator. Through the calculation of feature weight and the extraction of text features of keywords, the accuracy and efficiency of keywords are improved. Then, based on the matrix vector multiplication model of MapReduce, the keywords of cross-media writing text are obtained.

Secondly, when calculating the emotional intensity of the cross-media writing text, firstly, based on the existing emotional dictionary, an emotional dictionary containing emotional polarity and emotional intensity is constructed, including the dictionary of modifiers. Then, based on the constructed emotional dictionary, the emotion of the text is displayed. Trend calculation mainly constructs public opinion index model from objective and subjective aspects. Finally, the emotional intensity of writing text is analyzed through cross-media writing text, and the experimental results show that the model has certain reference function. In the future, with the upgrading of the system, the emotion recognition method will become more scientific and intelligent, but the operating steps of the current emotion recognition technology still need to be simplified.

## Data Availability Statement

The raw data supporting the conclusions of this article will be made available by the authors, without undue reservation.

## Ethics Statement

The studies were reviewed and approved by the Ethics Committee of East China Normal University. The participants provided their written informed consent to participate in this study.

## Author Contributions

RR designed the whole algorithm and experiments.

## Conflict of Interest

The author declares that the research was conducted in the absence of any commercial or financial relationships that could be construed as a potential conflict of interest.

## Publisher’s Note

All claims expressed in this article are solely those of the authors and do not necessarily represent those of their affiliated organizations, or those of the publisher, the editors and the reviewers. Any product that may be evaluated in this article, or claim that may be made by its manufacturer, is not guaranteed or endorsed by the publisher.
